# Behaviors of Shelter Dogs During Harnessing and Leash Walks: Prevalence, Demographics, and Length of Stay

**DOI:** 10.3390/ani15060856

**Published:** 2025-03-17

**Authors:** Betty McGuire, Bailey Guy, Miles Garland, Alexandra Jackson

**Affiliations:** 1 Department of Ecology and Evolutionary Biology, Cornell University, Ithaca, NY 14853, USA; msg284@cornell.edu; 2 Department of Animal Science, Cornell University, Ithaca, NY 14853, USA; bjg224@cornell.edu (B.G.); aej34@cornell.edu (A.J.)

**Keywords:** jumping, mouthing, leash-biting, pulling on leash, barking, sex, age, body size

## Abstract

Length of stay is an important metric for animal shelters, and studies have identified physical characteristics of dogs, and some behaviors, that predict length of stay. Although many shelters include leash walks when introducing dogs to potential adopters, it is unknown whether behaviors during harnessing and walking influence length of stay. During 707 walks of 120 dogs at a New York shelter, we recorded excitable behaviors, such as jumping on handlers, grabbing the leash, and pulling on the leash, whose measures of prevalence were largely unknown for shelter dogs. We also examined whether dog demographic characteristics predicted behaviors and whether behaviors predicted length of stay. During harnessing, jumping on handlers had the highest prevalence (45%), while during walking, pulling on the leash was most prevalent (86%). Dog age was the most common demographic predictor of behaviors, with jumping on handlers and total pulls/walk decreasing with age. Grabbing the leash predicted length of stay, with longer stays for dogs that displayed this behavior. These two findings suggest that it would be beneficial for shelter staff and volunteers to focus training efforts on younger dogs and those that grab the leash.

## Simple Summary

Length of stay at animal shelters is defined as time from an animal’s intake to its outcome. Studies have identified physical characteristics of dogs, and some behaviors, that predict length of stay. Leash walks with potential adopters are common when meeting a shelter dog, yet it is unknown whether behaviors shown during harnessing and walking influence length of stay. We observed 120 dogs during 707 walks at a New York shelter, focusing on excitable behaviors such as jumping on handlers, grabbing walking equipment, and pulling on the leash. We examined prevalence of behaviors, whether dog demographic characteristics predicted behaviors, and whether behaviors predicted length of stay. Jumping on handlers was the most prevalent behavior during harnessing (45%) and pulling on the leash during walking (86%). Age was the most common demographic predictor of behaviors, with younger dogs more likely than older dogs to jump on handlers and pull more frequently during walks. Grabbing the leash during a walk predicted length of stay: dogs showing this behavior had longer stays. Our data suggest that shelter staff and volunteers should consider focusing training efforts on younger dogs, and especially those that grab the leash during walks.

## 1. Introduction

Animal shelters are challenging environments for dogs due to stressors such as limited exercise and space, and exposure to high noise levels, unfamiliar people, and unfamiliar dogs [1,2]. These stressors can affect shelter dog behavior, physiology, and overall welfare [1]. Length of stay, measured as time elapsed from a dog’s intake to its outcome, is therefore a critical metric for shelters, affecting resident dogs as well as shelter operations, such as population management and staff workload.

Given the importance of length of stay to dog welfare and shelters, considerable research has examined factors that might influence this metric. Most research has focused on whether dog phenotypic and demographic characteristics, such as breed, body size, coat color and length, sex, and age, predict length of stay [3,4,5,6,7,8,9]. Other studies have examined whether dog behavior directly observed during shelter behavioral evaluations [10,11], in the kennel [6,12,13], or during interactions with potential adopters in out-of-kennel enclosed areas [14] predicts length of stay. Although leash walks often are part of dog meetings with potential adopters, to our knowledge, the question of whether dog behaviors during harnessing and walking influence length of stay has not been examined.

Dogs in homes and shelters sometimes display undesirable behaviors when meeting or greeting people and during harnessing and leash walking. Some of the most frequently reported problematic behaviors at these times include jumping on the handler, mouthing the handler, grabbing the harness or leash, and pulling on the leash [15,16,17,18,19,20,21,22,23,24]. Excessive barking is another unwanted behavior that can occur during leash walks, and in many other situations as well [17,25,26,27]. Problematic behaviors can result in relinquishment of dogs to shelters, a decision by potential adopters not to adopt after meeting a particular dog, and return of a dog to the shelter post-adoption [14,25,28,29,30].

In this study, conducted at a New York animal shelter, we examined whether behaviors displayed by 120 dogs during meeting/harnessing and leash walking predicted their length of stay. We focused on the behaviors jumping on the handler, mouthing the handler, grabbing walking equipment, pulling on the leash, and vocalizing. Given that these behaviors are frequently reported as undesirable in dogs [15,16,17,18,19,20,21,22,23,24], we predicted that dogs displaying them at our study shelter would have longer lengths of stay than dogs not showing them. Understanding the relationship between length of stay and behavior during an activity often included in meetings with potential adopters could inform shelter training and management of dog populations. A second objective was to determine prevalence of these behaviors in shelter dogs, because almost all studies on prevalence concern dogs living in homes; an exception is the prevalence of jumping/mouthing among shelter dogs reported by Marder et al. [22]. A third objective was to determine whether dog characteristics (sex, age, and body size) predict the display of these behaviors in shelter dogs. Although results vary by study and sometimes characteristic, the most commonly reported pattern for shelter and pet dogs is for undesirable behaviors, such as mouthing and pulling, to be more common in young dogs, so we expected to find this pattern in our data as well [20,21,31]. Almost all studies on prevalence and whether dog characteristics predict the behaviors we studied are based on owner-completed questionnaires or interviews; an exception is the work by Shih et al. [31], who used a leash tension meter to measure pulling in shelter dogs. In contrast to studies of pet dogs, our data are from direct behavioral observations of dogs.

## 2. Materials and Methods

Cornell University’s Institutional Animal Care and Use Committee approved all procedures outlined in our research protocol 2012-0150.

### 2.1. Study Shelter and Period

We collected behavioral data during harnessing and leash walks of dogs at the Tompkins County SPCA in Ithaca, NY, USA. The shelter is open-admission with scheduled intake. Adoption counselors meet with potential adopters using a conversation-based (rather than policy-based) approach. There are active volunteer programs for both cats and dogs. Volunteers working with dogs perform the following activities: training, socializing, leash walking, bringing dogs to the outdoor play yard, and occasionally bathing. All authors (three females and one male) volunteered in the dog wing at the shelter (three for 2–3 years each and the first author for 12 years). Formal data collection began in Fall 2023 and ended in Fall 2024; dog walks occurred in all intervening months.

### 2.2. Care and Housing of Dogs

Intake of dogs occurs in the Rescue Building, where they undergo a veterinary exam (vaccinations, fecal exam, deworming, flea control, and heartworm testing); a complete blood count/chemistry profile is run for older dogs. Dogs receive a microchip, if they lack one. Following the intake exam, dogs are housed in chain link cages with an indoor space (2.2 m^2^) and an outdoor run (3.5 m^2^).

Dogs are behaviorally evaluated about three days after intake in the Pet Adoption Center, which is connected to the Rescue Building via a walkway. Evaluations are conducted by two members of the behavior staff (one evaluator and one scribe), typically last about 30 min, and include a variety of tests and subtests [10,32,33]. Dogs are assigned color codes based on history reported by owners or finders, results of the behavioral evaluation, and age: yellow (shy dogs of any age); orange (older dogs without behavioral issues); green (young, energetic dogs); lilac (dogs of any age with mild to moderate behavioral issues, such as fear of strangers); and purple (dogs of any age with more severe behavioral issues, such as human-directed aggression). Dog codes can change based on observations and events following placement on the adoption floor; we report the final codes below (Section 2.3). Depending on level of experience and training at the shelter, volunteers are categorized as green walkers (can walk yellow, orange, and green dogs); lilac walkers (can walk yellow, orange, green, and lilac dogs); and purple walkers (can walk all dogs). The level of dog walker for each author was as follows: BM (purple); BG (lilac); MG (green); and AJ (green).

After behavioral evaluation, dogs move to the Pet Adoption Center, where they are housed in cubicles (from 5.2 to 7.3 m^2^) containing a bed, blanket, water bowl, toys, and often a crate. All dogs wear neck collars (buckle or martingale), and outside each dog’s cubicle hangs a harness and leash (at least 1.8 m long). Harnesses, fitted by staff, are usually the PetSafe^®^ EasyWalk^®^ brand (Radio Systems^®^ Corporation, Knoxville, TN, USA). Of the 120 dogs observed, only one wore a head collar, neck collar, and a harness. Feeding by staff occurs daily between 08:00 and 09:00 h and between 14:30 and 15:00 h.

Staff and volunteers exercise dogs several times a day. These sessions, which are recorded daily in the dog wing, include either leash walking or spending time in a large outdoor play yard. Additional forms of enrichment include overnight toys stuffed with food, pairs of compatible dogs either walked together or placed in the play yard together, and time spent away from the shelter with volunteers on either day outings or overnight stays. Almost all dogs are individually housed in both the Rescue Building and the Pet Adoption Center. Exceptions are made for puppies from the same litter and dogs from the same household that staff judge would benefit from pair housing.

### 2.3. Procedures

All authors individually walked dogs and collected behavioral data at the shelter from two to four times a week. Two-hour volunteer dog walking shifts occur at 12:00 and 14:30, and each Thursday, there is an additional shift at 17:00 h. All data for our study were collected during 12:00 shifts. Data collection began when a walker entered a cubicle and greeted and harnessed the dog (hereafter considered together as “harnessing”); this interaction was not timed, but we did document behaviors of interest. Upon exiting the shelter, we began data collection for the first 10 min of the leash walk; this interaction was precisely timed. Walks extended from shelter grounds to a field across the street (16.6 ha; 42°28′20″ N, 76°26′22″ W). The field was mostly grass; other fields, some of which were Cornell research plots, bordered the walking area as well as a creek and forest. We let dogs set the pace of walks and freely investigate their surroundings, but they were not allowed to directly interact with other dogs or people. Both in the cubicle and outside the shelter, we used our cell phones to verbally record data (e.g., the voice memo app on an iPhone 12, model MN9G2LL/A, Apple Inc., Cupertino, CA, USA), along with microphone-equipped Apple AirPods or similar devices. We photographed each dog, and walked and collected behavioral data on them for as long as they remained on the adoption floor, although new dogs were prioritized over dogs we had walked many times. Shelter staff prepare a daily treat bag for each dog on the adoption floor; we used treats when first entering a dog’s cubicle and when leaving the dog’s cubicle after a walk.

During harnessing and walking, we recorded each occurrence of the following five behaviors: jumping on the handler—dog’s front paws leave the ground and at least one contacts the handler [15]; grabbing the harness or leash—dog uses its teeth to seize walking equipment; when the leash is involved, often referred to as “leash biting” [24]; mouthing the handler—dog touches the handler’s clothing or skin with its teeth (modified from [20]); vocalizing—barks, growls, or whines; and pulling on the leash—leash becomes tight and the handler is visibly pulled by the dog [14]. Whereas jumping on the handler, grabbing walking equipment, mouthing, and vocalizing could occur both in the cubicle during harnessing and outside during walks, pulling on the leash only occurred during walks. Whenever possible, we categorized objects or individuals that dogs barked at or pulled towards (referred to as “targets”), as follows: dog with another person (e.g., volunteer walking another dog), another person (e.g., visitor to the shelter), parked or moving vehicle (e.g., car, truck, motorcycle, bicycle), and other (e.g., Canada geese in the field). For pulling on the leash, smell was also a target, scored when the dog pulled toward an object or location and then stopped to sniff it. If vocalizing and pulling occurred with no apparent target, we recorded “no target”.

We transferred data from verbal recordings of behaviors displayed during harnessing and the first 10 min of walks onto paper check sheets. These sheets also included the walker’s name and the following information for each dog: name, identification number, sex, age, body mass, spay/neuter status, source, color code from behavioral evaluation, and walk number. We collected data on 124 dogs, but excluded data from the following four dogs: two juveniles that were housed in the same cubicle and had to be walked together, and one juvenile and one adult that required major surgeries and spent long periods of time recuperating in foster homes. Length of stay at our study shelter is longer for dogs in foster homes than for dogs housed at the shelter [10]. This left 120 dogs for data analyses (9 yellow; 10 orange; 33 green; 54 lilac; and 14 purple). Total number of walks per dog ranged from 1 to 31 (*median*, 4; *mean*, 5.9; *SD*, 5.3) and total number of walks for the 120 dogs was 707. The number of observers that individually walked each dog ranged from 1 to 4, with a median of 1 (1 observer, 83 dogs; 2 observers, 22 dogs; 3 observers, 9 dogs; 4 observers, 6 dogs).

We assigned dogs to the following age classes: juvenile (from 4 months to <1 year); younger adult (from 1 to 3 years); older adult (>3 years to <7 years); and senior (≥7 years). Body size categories included small (<11 kg), medium (11–24 kg), and large (≥25 kg). Most dogs are spayed or neutered before arriving on the adoption floor; all are spayed or neutered before entering adoptive homes. Of the 120 dogs walked, 42 of 46 females were spayed for all of their walks and 66 of 74 males were neutered for all of their walks. One female was intact for her first and only walk, two females were intact for their first or first and second walks and spayed for subsequent walks, and another was intact for her first five walks and spayed for subsequent walks. Five males were intact for their first or first and second walks and neutered for subsequent walks, and three were intact for their first two or three walks. Sources of dogs observed included surrender by owner, transfer from another shelter, seizure by animal control officers, and pick-up as a stray. Table 1 summarizes demographic and phenotypic characteristics of the dogs by sex.

Finally, we retrieved data entered by shelter staff into the PetPoint data management system; such data included intake date, outcome date, length of stay (for our study in which all 120 dogs were adopted, defined as date adoption paperwork signed minus intake date, in days), and whether the dog was returned to the shelter. For dogs returned to the shelter, we used their first length of stay and behavioral data during that stay in statistical analyses. We uploaded all data to Box, a service for data and document sharing and storage.

### 2.4. Statistical Analyses

We used JMP Pro version 17.0.0 (© 2022, JMP Statistical Discovery, LLC, Cary, NC, USA) to calculate prevalence of behaviors. All other analyses were performed in R version 4.4.0 [34]. Statistical significance was set at *p* ≤ 0.05.

For all analyses, we separated behaviors during harnessing and walking. Four behaviors were displayed by only a few dogs or by less than half of the dogs: jumping on the handler, grabbing walking equipment, mouthing the handler, and vocalizing. We converted our count data for these four behaviors to binary data (displayed; 0 = no; 1 = yes). Pulling on the leash was displayed by most dogs, so we left these data as counts, and also calculated inter-observer reliability for this behavior. From a set of 12 videotapes, we randomly selected 4. All authors scored number of pulling incidents for these videotaped walks of four different dogs. An intra-class correlation coefficient (ICC) was estimated as 0.90 using R package irr [35] with a 95% CI of (0.63, 0.99). The ICC was significantly greater than zero (*p* < 0.001), indicating good inter-observer reliability for this behavior.

#### 2.4.1. Prevalence of Behaviors

We calculated prevalence of each behavior during either harnessing or walking as the number of dogs showing the behavior at the time of their first walk/120 dogs. When dogs were walked by more than one member of the research team, we used the first walk with the earliest date. We followed the same procedures for vocalizing at and pulling toward specific targets.

#### 2.4.2. Dog Characteristics and Behaviors

Data from all 707 walks were used for these analyses. We summarized data into percentages and frequencies for categorical data (jumping on handler, grabbing walking equipment, mouthing handler, and vocalizing) and means and standard deviations for continuous data (total pulls/walk). Generalized linear models with a binomial distribution were used to model binary behaviors as a function of dog sex, age class, body size, and walk number with random effects of dog ID and walker ID. A generalized linear model with a negative binomial distribution was used to model total pulls during a walk (data were very skewed to the right) as a function of dog sex, age class, body size, and walk number with random effects of dog ID and walker ID. We did not model rare behaviors (i.e., those displayed by <5% of dogs). We conducted post hoc pairwise comparisons with a Tukey correction for significant main effects for each model. R package lme4 [36] was used for generalized linear models and package emmeans for post hoc tests [37].

#### 2.4.3. Dog Characteristics, Behaviors, and Length of Stay

For the length-of-stay analysis, we used data from each dog’s first walk because dogs with longer lengths of stay would likely have more walks and therefore more opportunities to display the behaviors monitored. We ran a multivariate linear model to examine the log of the length of stay as a function of dog characteristics (sex, age class, and body size), as well as the behavior variables (categorical variables for jumping on handler during harnessing and walking, grabbing walking equipment during walking, and vocalizing during walking; and the continuous variable, total number of pulls during the walk). Mouthing during harnessing and walking, as well as grabbing walking equipment and vocalizing during harnessing, were not included because they were displayed by <5% of dogs. We conducted post hoc pairwise comparisons with a Tukey correction for significant effects, using R package emmeans [37].

## 3. Results

### 3.1. Prevalence

Table 2 shows prevalence of behaviors during first walks. During harnessing, prevalence was highest for jumping on the handler, with nearly half of dogs showing this behavior; other behaviors were shown by only a few dogs. During walking, prevalence was highest for pulling on the leash, with a majority of dogs showing this behavior, followed by jumping on the handler and vocalizing; few dogs grabbed the leash, and even fewer mouthed the handler.

Of the 26 dogs that vocalized during walking, 21 barked, 3 whined, 1 barked and whined, and 1 barked and growled. Targets of vocalizations during leash walks were as follows (note that these do not equal 26, because some dogs vocalized at more than one target during their walk): dog with another person, 14.2% (17/120); person, 5.0% (6/120); vehicle, 2.5% (3/120); other, 1.7% (2/120); and no apparent target, 4.2% (5/120). Targets for pulling on the leash were as follows (targets do not equal 103, the number of dogs that pulled on the leash, because many dogs pulled toward more than one type of target during their walk): dog with another person, 23.3% (28/120); person, 8.3% (10/120); vehicle, 6.7% (8/120); smell, 54.2% (65/120); other, 8.3% (10/120); and no target, 66.7% (80/120).

### 3.2. Dog Characteristics and Behavior

Descriptive statistics based on raw data for behaviors in relation to age class are shown in Table 3 and in relation to sex and body size in Table 4.

#### 3.2.1. Jumping on Handler

Dogs jumped on the handler during 300 of the 707 harnessing sessions (42.4%). We found a significant effect of age class for this behavior (*X*^2^ = 32.7, *d.f.* = 3, *p* < 0.001). Pairwise comparisons revealed that the odds of jumping while being harnessed were higher for juveniles (predicted probability of 0.901) as compared to younger adults (predicted probability of 0.464; *p* < 0.01), older adults (predicted probability of 0.079; *p* < 0.001), and seniors (predicted probability of 0.042; *p* < 0.001). Younger adults also jumped more than older adults (*p* < 0.01) and seniors (*p* < 0.05). We did not find significant effects of sex (*X*^2^ = 3.5, *d.f.* = 1, *p* = 0.06), body size (*X*^2^ = 4.0, *d.f.* = 2, *p* = 0.13), or walk number (*X*^2^ = 1.5, *d.f.* = 1, *p* = 0.22).

Dogs jumped on the handler in 109 of 707 leash walks (15.4%). No seniors displayed this behavior (Table 3), so this age group was removed from the model. We found a significant effect of age class for jumping during walking (*X*^2^ = 14.5, *d.f.* = 2, *p* < 0.001). The odds of jumping were higher for juveniles (predicted probability of 0.186) when compared to older adults (predicted probability of 0.004; *p* < 0.01), and for younger adults (predicted probability of 0.060) when compared to older adults (predicted probability of 0.004; *p* < 0.05). Post hoc tests run to see whether the predicted probabilities of juveniles, younger adults, and older adults were significantly different from zero (seniors; using a logit of −7 to approximate zero) revealed that juveniles and younger adults were significantly more likely than seniors to jump on the handler during walks (*p* < 0.001 for both comparisons). We also found a significant effect of walk number (*X*^2^ = 7.7, *d.f.* = 1, *p* < 0.01), with the odds of jumping on the handler decreasing by about 16% with each subsequent walk. We did not find significant effects of sex (*X*^2^ = 0.4, *d.f.* = 1, *p* = 0.55) or body size (*X*^2^ = 3.1, *d.f.* = 2, *p* = 0.22).

#### 3.2.2. Grabbing Walking Equipment

Dogs grabbed the harness in 15 of 707 harnessing sessions (2.1%); dogs showing this behavior were either juveniles or younger adults (Table 3) and of either medium or large body size (Table 4). Grabbing the harness was not modeled due to its rarity.

Dogs grabbed the leash in 31 of 707 walks (4.4%); dogs displaying this behavior were either juveniles or younger adults (Table 3). Grabbing the leash was not modeled due to its rarity.

#### 3.2.3. Mouthing Handler

When being harnessed, dogs mouthed the handler in 27 of 707 sessions (3.8%), and during walks, in 19 of 707 (2.7%). Mouthing during harnessing and walking was exhibited only by juveniles and younger adults (Table 3), and was not modeled due to its rarity.

#### 3.2.4. Vocalizing

During harnessing, 11 of 707 sessions (1.6%) included dogs that vocalized; dogs showing this behavior were all younger adults (Table 3) of medium to large body size (Table 4). Due to its rarity, vocalizing during harnessing was not modeled.

Vocalizing during walking was more common, occurring in 151 of 707 leash walks (21.4%). This behavior was displayed by all age classes (Table 3), both sexes, and all body sizes (Table 4). We found a significant effect of sex for vocalizing during leash walks (*X*^2^ = 3.9, *d.f.* = 1, *p* < 0.05), with the odds being higher for female dogs (predicted probability of 0.184) than for male dogs (predicted probability of 0.091). Effects of age class (*X*^2^ = 3.6, *d.f.* = 3, *p* = 0.31), body size (*X*^2^ = 1.7, *d.f.* = 2, *p* = 0.43), and walk number (*X*^2^ = 0.9, *d.f.* = 1, *p* = 0.34) were not significant.

#### 3.2.5. Pulling on Leash

**Figure 1:**
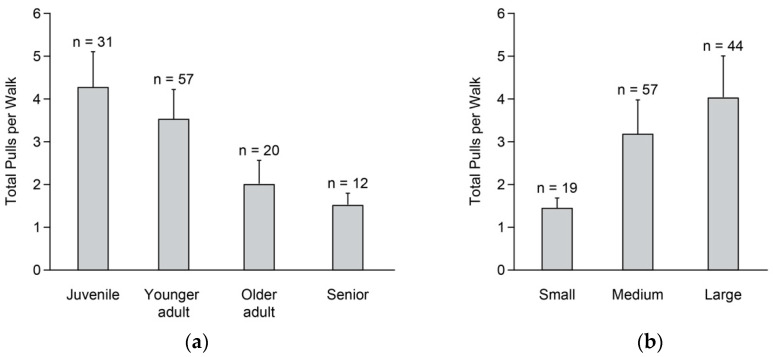
Predicted total pulls on the leash for the first 10 min of walks by shelter dogs in relation to age class (**a**) and body size (**b**). Total walks = 707. Juveniles, from 4 months to <1 year; younger adults, from 1 year to 3 years; older adults, from 4 to <7 years; and seniors, ≥7 years. Small, <11 kg; medium, 11–24 kg; and large, ≥25 kg.

Based on raw data, the average number of pulls per walk ± *SD* was 6.1 ± 6.3. We found a significant effect for age class (*X*^2^ = 12.0, *d.f.* = 3, *p* < 0.01), with the general pattern of total pulls/walk decreasing with age (Figure 1a). Juveniles pulled more than seniors (*p* = 0.02), and there were tendencies for juveniles to pull more than older adults (*p* = 0.07) and for younger adults to pull more than seniors (*p* = 0.08; Figure 1a). We also found a significant effect for body size (*X*^2^ = 12.0, *d.f.* = 2, *p* < 0.01): here, the general pattern was for total pulls/walk to increase with body size (Figure 1b). Medium dogs pulled more than small dogs (*p* = 0.02), and large dogs pulled more than small dogs (*p* < 0.01). Effects of sex (*X*^2^ = 0.1, *d.f.* = 1, *p* = 0.73) and walk number (*X*^2^ = 0.8, *d.f.* = 1, *p* = 0.38) were not significant.

### 3.3. Dog Characteristics, Behavior, and Length of Stay

**Figure 2:**
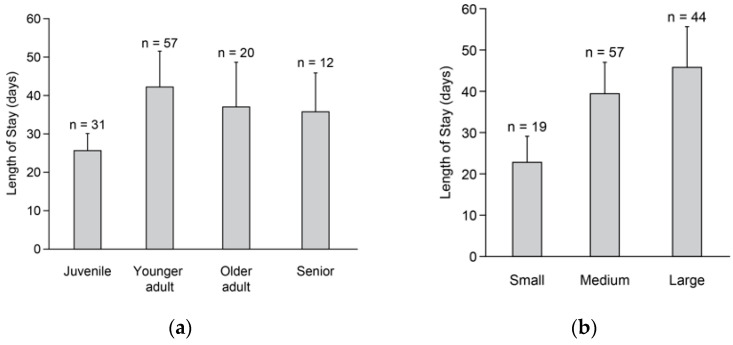
Predicted length of stay (in days) for 120 shelter dogs placed up for adoption in relation to age class (**a**) and body size (**b**). Juveniles, from 4 months to <1 year; younger adults, from 1 year to 3 years; older adults, from 4 to <7 years; and seniors, ≥7 years. Small, <11 kg; medium, 11–24 kg; and large, ≥25 kg.

Length of stay based on raw data in relation to dog characteristics is shown in Table 5. There was a tendency for age class to influence length of stay (*F* = 2.43, *df* = 3,108, *p* = 0.08), with younger adults having longer stays than juveniles (*p* = 0.05; Figure 2a). We found a significant effect of body size on length of stay (*F* = 3.21, *df* = 2,108, *p* = 0.05), with large dogs having longer stays than small dogs (*p* = 0.04; Figure 2b). Sex did not influence length of stay (*F* = 0.2, *df* = 1,108, *p* = 0.70).

Length of stay based on raw data in relation to whether behaviors occurred during harnessing or walking is shown in Table 6. 

**Figure 3:**
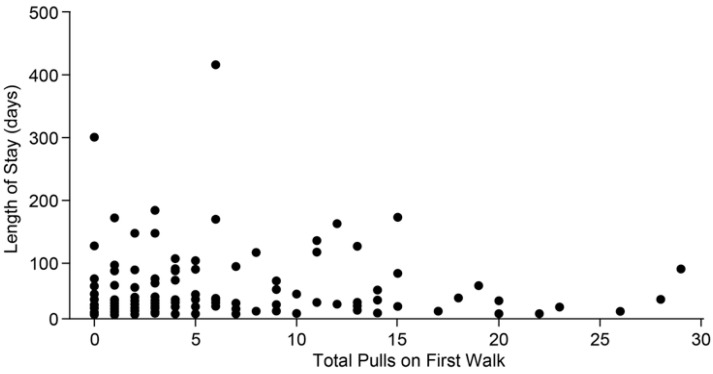
Length of stay (in days and from raw data) for 120 shelter dogs placed up for adoption in relation to total number of pulls during the first walk.

Four behaviors, three during harnessing (mouthing handler, grabbing walking equipment, and vocalizing) and one during walking (mouthing handler), were not included in the model because they occurred in <5% of dogs during first walks (Table 6). Of the remaining behaviors, grabbing walking equipment predicted length of stay (*F* = 4.0, *df* = 1,108, *p* < 0.05), with dogs that grabbed the leash during the first walk having longer stays (emmean ± *SE*, 47.3 ± 15.4 days) than those that did not (24.7 ± 3.4 days). None of the other binary behaviors during either harnessing (jumping on handler: *F* = 0.8, *df* = 1,108, *p* = 0.39) or walking predicted length of stay (jumping on handler: *F* = 0.02, *df* = 1,108, *p* = 0.89; vocalizing: *F* = 0.01, *df* = 1,108, *p* = 0.93). Figure 3 shows total pulls during the first walk based on raw data in relation to length of stay. Total pulls did not predict length of stay (*F* = 0.3, *df* = 1,108, *p* = 0.59).

## 4. Discussion

In this study, we provide new data on the behaviors of shelter dogs during routine, daily activities. Our measures of prevalence were derived from dogs’ first walks and included behaviors displayed during harnessing, which also included meeting the dog for the first time in its cubicle, and leash walking. We found jumping on the handler to be the most prevalent behavior during harnessing and pulling on the leash during walking. Dog age was the most common demographic predictor of behaviors, with jumping on the handler during both harnessing and walking and pulling on the leash decreasing with age. Grabbing the leash during a walk predicted length of stay at the shelter: dogs that displayed this behavior had longer stays than those that did not.

Given the limited literature on shelter dogs for our behaviors of interest, most of our comparisons with other studies concern data collected on pet dogs; these are not direct one-to-one comparisons for several reasons. The investigations with pet dogs differed from ours not only in study population, but also in methodology: whereas studies of dogs in homes used owner-completed questionnaires, we conducted direct behavioral observations. Also, due to the difference in environments experienced by pet dogs and shelter dogs, their relationships and level of familiarity with handlers are very different. For these reasons, we cautiously make the below comparisons and always specify the type of study.

### 4.1. Prevalence

During harnessing, prevalence was highest for jumping on the handler (45%), with other behaviors displayed by less than 4% of dogs (grabbing walking equipment, mouthing the handler, and vocalizing). Our prevalence for jumping on the handler during harnessing can be compared to several prevalence measures for jumping on handlers from studies based on owner-completed questionnaires that examined situations similar to our own: 46% of dogs frequently or always jumped on owners when they picked up the leash to take their dog outside [18]; 73% of dogs, at some point in their lifetime, jumped on a household member before going for a walk [15]; and 59% of dogs jumped on a stranger entering their home [16]. Finally, Polian et al. [15] found that jumping on household members before going for a walk was often preceded by canine care-seeking behaviors, such as approaching in a lowered posture with hindquarters wagging, and was more common in dogs walked less than once a day compared to those walked two or more times a day, suggesting that when dogs jump on people, they are conveying information about their needs. In the shelter environment, as in a home, these needs would include going outside for elimination, exercise, and cognitive stimulation.

During walking, prevalence was highest for pulling on the leash, followed by jumping on the handler and vocalizing; relatively few dogs grabbed the leash or mouthed the handler. Our prevalence for pulling on the leash (86%) is similar to the 83% reported by one study based on owner-completed questionnaires [38] and somewhat higher than the 69% reported by another [17]. The most common targets to which dogs pulled in our study included no apparent target, a smell, or a dog walking with another person. Our prevalence for jumping on the handler during the first walk (24%) was similar to that reported by Rezac et al. [16] for dogs jumping on strangers during a walk (27%). Our overall prevalence for vocalizing during a leash walk (22%) is somewhat higher than the 12% reported by Dinwoodie et al. [27] for excessive barking by pet dogs at triggers when outside. We found that about 14% of dogs barked at a dog walked by another person, which is somewhat lower than the 26% of owners reporting that their dog barks aggressively at other dogs on a leash [18].

Whether during harnessing (3.3%) or walking (2.5%), our prevalence measures for mouthing the handler were lower than reported in studies based on owner-completed questionnaires: 45% of respondents said that their dog currently mouthed and 21% previously mouthed [20]; 37% of dogs between 11 and 18 months old mouthed [39]; and when owners picked up the leash, 12 to 13% of dogs frequently or always mouthed causing no discomfort, 3% causing discomfort, and 6% grabbing clothing [18]. Although the functions of mouthing are somewhat unclear, more than half of owners reported that their dog mouthed during play and when in the presence of an exciting stimulus, such as the owner returning home or a guest entering the home [20]. Additionally, a study with three pet dogs suggested that dogs mouth either to get their owner’s attention or gain access to tangibles [40], which could include treats, toys, or walks. To our knowledge, the only other report on mouthing by dogs in shelters combined mouthing and jumping (37%) [22]. This makes it challenging for us to put our results in context to determine whether the difference between our findings and those from dogs in homes reflects the lack of a relationship between us and the dogs when we first met, harnessed, and walked them. Some excitable behaviors are more commonly directed at household members than strangers: for example, dogs were eight times more likely to jump on a household member entering their home than a stranger [16]. It would be interesting to know whether, as with jumping on someone, dogs are more likely to mouth household members than strangers, but to our knowledge this question has not been examined.

### 4.2. Dog Characteristics and Behavior

We found that dogs jumped on the handler in 42% of harnessing sessions and 15% of leash walks. This pattern is similar to that found by Rezac et al. [16]: dogs were four times more likely to jump up on a stranger entering the home than a stranger while on a leash walk, and twelve times more likely to jump on a household member entering the home than a household member on a leash walk. For jumping on a handler both during harnessing and walking, we found significant effects of age class, with jumping decreasing with age. Although jumping on people is commonly believed to be associated with dogs of younger ages, our findings may be the first to actually document this pattern. We found no effects of dog sex or body size for jumping on handlers, which is consistent with findings for dogs in homes [15]. Finally, the odds of a dog jumping on the handler decreased with each subsequent walk. One possible explanation for this decrease over time is that dogs became more accustomed to the walking routine and schedule at the shelter and felt less of a need to convey what they wanted or needed.

During both harnessing and walking, mouthing the handler could not be modeled, because the behavior occurred in less than 5% of walks, as did grabbing walking equipment during harnessing and walking. Nevertheless, our descriptive data showed that all four of these behaviors only occurred in juveniles and younger adults, suggesting that they might also decrease with age. This pattern is similar to that found by Waite et al. [20] for dogs reported by their owners to currently mouth: 80% of dogs < 6 months of age; 83% of dogs from 6 to 11 months of age; 40% of dogs ≥ 12 months of age; and 26% of dogs > 5 years old.

Although vocalizing during harnessing occurred very rarely and therefore could not be modeled, vocalizing during walking was more common and could be modeled. We found a significant effect of sex, with the odds of vocalizing during a leash walk (almost all vocalizations were barks) being higher for females than males. Of three studies based on owner-completed questionnaires, one found that female dogs were more likely than males to exhibit excessive barking [41], whereas the other two found no sex difference [42,43].

Age class predicted total pulls on the leash, with a decrease with age as the general pattern (juveniles pulled more than seniors, and there were tendencies for juveniles to pull more than older adults and younger adults to pull more than seniors). We also found a significant effect of body size, with medium and large dogs pulling more frequently than small dogs. Dog sex and walk number did not affect total pulls on the leash in our study. Shih et al. [31] also found that younger dogs pulled more than older dogs; however, larger, heavier dogs, despite exerting higher leash tensions, pulled less frequently than smaller, lighter dogs in their study. In a separate paper, Shih et al. [44] reported that male dogs exerted higher leash tensions and pulled more frequently than did female dogs. It is possible that some of the differences in findings between Shih’s two studies and our study reflect differences in how pulling was assessed and defined: whereas Shih et al. [31,44] used a leash tension meter and defined a pull event as when the leash tension suddenly spiked above baseline tension, we scored pulling as when the leash became tight, and the handler was visibly pulled by the dog.

### 4.3. Dog Characteristics, Behavior, and Length of Stay

There was a tendency for a dog’s age to predict length of stay in our study; the lack of an overall significant effect of age may reflect our relatively small sample sizes for older adults and especially seniors. In pairwise comparisons, however, younger adults did have significantly longer stays than juveniles. In a previous study at this shelter, with a much larger sample size (975 dogs) and only three age classes rather than four, seniors had longer lengths of stay than adults, which in turn had longer lengths of stay than juveniles [10]. Increases in length of stay with age have been reported by others [4,7,8,11]; one exception concerns a study that did not include dogs over 7 years old [45].

Body size predicted length of stay at our shelter, with large dogs having longer stays than small dogs. Mesarcova et al. [11] also found longer lengths of stay for larger dogs, and Brown et al. [8] and Žák et al. [7] found that both medium and large dogs had longer stays than small dogs. Interestingly, Žák et al. [7] also reported that giant dogs (>65 cm at withers) had the shortest length of stay of all size groups, possibly because they were so unusual in the three Czech shelters studied (only seven of the 2261 dogs abandoned and put up for adoption). Our sample size was too small to permit further differentiation of size classes.

Of the behaviors studied, only grabbing the leash during the first walk predicted length of stay, with dogs displaying this behavior remaining longer at the shelter than those that did not. The only research papers that we found on leash-biting concerned its display in response to either different head collars [46] or head versus neck collars [47], and only one of the 120 dogs in our study wore a head collar, neck collar, and harness. Leash-biting, along with several other behaviors studied here—jumping up, mouthing, and pulling on the leash—are often described as “excitable behaviors” [18]. However, it remains somewhat unclear whether this categorization is accurate and how these behaviors might relate to one another or to other behaviors [18]. Additionally, while progress has been made in identifying the conditions under which jumping up [15,16,18], mouthing [18,20], and leash-pulling occur [48], this is not the case for leash-biting, which seems unstudied outside its connection with different types of collars [46,47]. Understanding the conditions in which leash-biting occurs could help shelter staff and volunteers mitigate this behavior.

In a previous study at our shelter, performance on two of 13 tests/subtests of the behavioral evaluation predicted length of stay, with longer stays for dogs displaying either severe food guarding, when either a bowl of food was removed during the food bowl test and/or a treat was taken away during the possession test, or dangerous behavior when leashed and meeting another dog, also on a leash [10]. Adoption counselors would certainly discuss these behaviors with potential adopters, which may have discouraged some (e.g., those with young children or a resident dog), leading to a smaller pool of potential adopters and longer stays. Although some of the behaviors studied here, such as jumping on handlers, mouthing, and pulling on the leash, can pose risks to humans [16,18,49], the risks associated with these excitable behaviors would seem lower than those associated with either severe food aggression or dog-directed aggression (e.g., when owners try to break up dog fights); this might explain our failure to find a relationship between jumping up and pulling on the leash and length of stay (mouthing was too rare to include in the length-of-stay model). It is also possible that some adopters view jumping on people as a friendly behavior [50]. Finally, jumping on the handler is amenable to training, and selection of appropriate walking equipment can help mitigate pulling on the leash [23,51]; some potential adopters may be aware of this information.

When compared to demographic or phenotypic characteristics, which often predict length of stay and adoption decisions for shelter dogs (reviewed in [52]), relatively few behavioral characteristics are predictive. For example, Mesarcova et al. [11] found that age and body size affected length of stay, but none of the four categories of behaviors evaluated by shelter staff influenced time spent at the shelter. Protopopova et al. [12] reported that only three of 41 in-kennel behaviors, scored and found to have high inter-observer reliability, predicted length of stay, with longer stays for dogs that stood, faced away from the front of the kennel, or leaned or rubbed on kennel walls. In another in-kennel study, dogs with higher frequencies of inner brow raises, a movement that makes the eyes appear larger in relation to the face (i.e., a pedomorphic feature), had shorter stays than dogs with lower frequencies [13]. Frequencies of eight other facial movements were not associated with length of stay, and durations of tail wagging and spending time in close proximity to the experimenter were weakly associated, but in different ways: whereas more time spent wagging the tail was associated with longer stays, more time spent in close proximity to the experimenter was associated with shorter stays [13]. Some of the behaviors that we studied (mouthing, jumping up, barking, and on-leash pulling), along with 25 other behaviors, were examined with respect to adopter decisions after meeting dogs in enclosed areas away from their kennels, and only two behaviors influenced decisions: dogs that were adopted after the meeting spent less time ignoring play initiations by and more time lying in proximity to potential adopters [14]. Finally, Weiss et al. [50] found that adopters of dogs at five different shelters listed physical appearance as the single most important factor in their choice of dog (27%), followed by personality/temperament (16%) and behavior with people (11%). In total, these results suggest that dog physical characteristics more routinely predict actual adoption than do most behaviors.

### 4.4. Study Limitations

Our study has several limitations. First, we had a relatively small sample size (120 dogs), especially with respect to older adults and seniors, and small dogs. Second, walkers could not be randomly assigned to dogs due to the necessary walking regulations at the shelter whereby level of walker experience was matched, using the color coding system, with the level of behavioral challenges of dogs. Third, our results are from one shelter and may not generalize to other shelters. In particular, the amount of time that dogs are outside at our study shelter may differ from that at other shelters, and increased exercise frequency and duration are associated with lower levels of excitable behaviors, at least in pet dogs [15,53]. For this reason, we further detail time outside at our study shelter here. When Animal Care Technicians arrive in the morning, all dogs are given a few minutes each outside in a covered play yard. Then, while their cubicle is cleaned, the dogs are cycled into one of three locations, two indoor vestibules and the covered play yard, such that one third of dogs on the adoption floor also get about 20 min of outdoor time during cleaning. When the shelter opens at noon to the public and volunteers, dogs typically get three or four times outside before the shelter closes (either leash walks or time in the large outdoor play yard). Finally, our inter-observer reliability check for pulling on the leash was based on videotaped walks of only four different dogs. A larger number of dogs would have been preferable, because it would have allowed us to observe and score more of the variation in the dog population at our study shelter [54].

## 5. Conclusions

Although not directly comparable, given differences in study populations, methodologies, and degree of familiarity with handlers, our measures of prevalence were generally similar to or lower, in the case of mouthing the handler, than reported for dogs in homes. Dog age was the most common demographic predictor of behaviors with sufficient occurrence for us to model them, with jumping on the handler and total pulls on the leash decreasing with age. Consistent with these findings, our descriptive data for rare behaviors that could not be modeled (mouthing the handler and grabbing walking equipment) showed that these behaviors were displayed by juveniles and younger adults, but not by older adults and seniors, suggesting that these behaviors might also decrease with age. When looking at whether behaviors during the first walk influenced length of stay, grabbing the leash predicted time spent at the shelter: dogs that grabbed the leash had longer stays than those that did not. Our findings regarding dog age and grabbing the leash suggest that it might be beneficial for shelter staff and volunteers to focus training efforts on younger dogs, and especially those that display leash-biting. Leash-biting is an understudied behavior, and additional research is needed to better understand the conditions in which it occurs, which could aid efforts to mitigate this behavior.

## Figures and Tables

**Table 1:** Characteristics of the 120 shelter dogs included in our study.

Dog Characteristics	Females	Males
Age class ^1^		
Juvenile	10	21
Younger adult	25	32
Older adult	6	14
Senior	5	7
Size class ^2^		
Small	7	12
Medium	21	36
Large	18	26
Source		
Surrender by owner	24	43
Transfer from another shelter	3	5
Seizure by animal control officer	4	11
Pick-up as a stray	15	15

^1^ Juveniles, from 4 months to <1 year; younger adults, from 1 year to 3 years; older adults, from 4 to <7 years; and seniors, ≥7 years. ^2^ Small, <11 kg; medium, 11–24 kg, and large, ≥25 kg.

**Table 2:** Prevalence of behaviors during the first 10 min of first walks.

Behavior	During Harnessing	During Walking
Jumping on handler	45.0% (54/120)	24.2% (29/120)
Grabbing walking equipment	1.7% (2/120)	8.3% (10/120)
Mouthing handler	3.3% (4/120)	2.5% (3/120)
Vocalizing	2.5% (3/120)	21.7% (26/120)
Pulling on leash	-----	85.8% (103/120)

**Table 3:** Descriptive statistics based on raw data for behaviors in relation to dog age class. Categorical data are shown as percentages of dogs showing the behavior and continuous data as means and standard deviations.

Behavior	Juvenile ^1^	Younger Adult	Older Adult	Senior
Jumping on handler				
Harnessing	75.0	48.4	14.2	9.1
Walking	37.5	16.3	1.4	0.0
Grabbing walking equipment				
Harnessing	2.5	3.2	0.0	0.0
Walking	13.3	4.0	0.0	0.0
Mouthing handler				
Harnessing	10.8	3.7	0.0	0.0
Walking	6.7	2.9	0.0	0.0
Vocalizing				
Harnessing	0.0	2.9	0.0	0.0
Walking	22.5	24.7	8.5	27.3
Total pulls/Walk				
Walking	6.6 ± 6.9	6.4 ± 6.8	6.1 ± 5.2	3.4 ± 3.4

^1^ Juveniles, from 4 months to <1 year; younger adults, from 1 year to 3 years; older adults, from 4 to <7 years; and seniors, ≥7 years.

**Table 4:** Descriptive statistics based on raw data for behaviors that occurred during either harnessing or walking in relation to dog sex and body size. Categorical data are shown as percentages of dogs showing the behavior and continuous data as means and standard deviations.

	Sex		Size ^1^		
Behavior	Female	Male	Small	Medium	Large
Jumping on handler					
Harnessing	50.4	37.9	23.3	53.6	34.7
Walking	17.2	14.4	12.3	21.9	9.0
Grabbing walking equipment					
Harnessing	3.1	1.6	0.0	2.7	2.0
Walking	3.1	5.1	5.5	5.1	3.3
Mouthing handler					
Harnessing	6.2	2.4	2.7	4.5	3.3
Walking	2.3	2.9	2.7	3.9	1.3
Vocalizing					
Harnessing	3.5	0.4	0.0	0.6	3.0
Walking	27.7	17.7	34.2	24.6	14.7
Total pulls/Walk					
Walking	6.7 ± 7.0	5.8 ± 5.9	2.7 ± 4.1	6.7 ± 7.1	6.3 ± 5.6

^1^ Small, <11 kg; medium, 11–24 kg; and large, ≥25 kg.

**Table 5:** Length of stay (mean ± *SD*, in days) based on raw data for shelter dogs placed up for adoption in relation to sex, age class, and body size (sample sizes in parentheses).

Dog Characteristics	Length of Stay (Days)
Sex	
Female	54.3 ± 77.6 (46)
Male	44.2 ± 40.8 (74)
Age class ^1^	
Juvenile	24.9 ± 18.4 (31)
Younger adult	64.0 ± 74.5 (57)
Older adult	48.3 ± 42.0 (20)
Senior	32.1 ± 24.0 (12)
Size class ^2^	
Small	23.7 ± 20.2 (19)
Medium	52.7 ± 72.2 (57)
Large	52.7 ± 43.9 (44)

^1^ Juveniles, from 4 months to <1 year; younger adults, from 1 year to 3 years; older adults, from 4 to <7 years; and seniors, ≥7 years. ^2^ Small, <11 kg; medium, 11–24 kg; and large, ≥25 kg.

**Table 6:** Length of stay (mean ± *SD*, in days) based on raw data for shelter dogs placed up for adoption in relation to whether behaviors were shown (no/yes) during harnessing or walking (sample size in parentheses) for the first walk. * Indicates that <5% of dogs showed this behavior.

Behavior	Length of Stay (Days)
Jumping on handler	
Harnessing—no	54.3 ± 69.5 (65)
Harnessing—yes	40.7 ± 38.8 (55)
Walking—no	48.8 ± 51.4 (91)
Walking—yes	45.7 ± 75.0 (29)
Grabbing walking equipment	
Harnessing—no	48.6 ± 58.0 (118)
Harnessing—yes *	17.5 ± 3.7 (2)
Walking—no	45.1 ± 47.6 (110)
Walking—yes	81.1 ± 123.0 (10)
Mouthing handler	
Harnessing—no	45.6 ± 47.3 (116)
Harnessing—yes *	121.0 ± 197.0 (4)
Walking—no	48.4 ± 58.3 (117)
Walking—yes *	33.9 ± 21.1 (3)
Vocalizing	
Harnessing—no	48.9 ± 58.1 (117)
Harnessing—yes *	17.1 ± 8.7 (3)
Walking—no	50.0 ± 62.0 (94)
Walking—yes	41.1 ± 37.9 (26)

## Data Availability

All data are available online as Supplementary Materials.

## Supplementary Materials

The following supporting information can be downloaded at: https://www.mdpi.com/article/10.3390/ani15060856/s1, Table S1. Prevalence data for first walks.xlsx, Table S2. Dog demographics and behaviors for all walks.xlsx, and Table S3. Behaviors during first walks and length of stay.xlsx. The R scripts used in analyzing data are also included: R scripts for Dog Demographics and Behavior.doc and R scripts for Dog Behavior and Length of Stay.doc.
