# Supplementary material for: Behaviors of Shelter Dogs During Harnessing and Leash Walks: Prevalence, Demographics, and Length of Stay
Source: Animals (Basel). 2025 Mar 17;15(6):856. doi: 10.3390/ani15060856 (PMC11939286; doi:10.3390/ani15060856)
Supplement: Supplementary file 1 [file animals-15-00856-s001.zip › R scripts for Dog Behavior and Length of Stay.pdf]

```
#This R file looks at the length of stay and how that can be predicted by dog behaviors and demographics

#Libraries
library(lme4)
library(lmerTest)
library(emmeans)
library(ggplot2)
library(car)
library(dplyr)
library(survival)
library(effectsize)
library(survminer)
library(gtsummary)

LOS_dat <- read.csv("Length_of-Stay.csv")

#Predictor variables \ Characteristics
LOS_dat$Dog_ID<-as.factor(LOS_dat$Dog_ID)
LOS_dat$Sex<-as.factor(LOS_dat$Sex)
LOS_dat$Age_Class<-as.factor(LOS_dat$Age_Class)
LOS_dat$Body_Size<-as.factor(LOS_dat$Body_Size)
```

```
##SETTING UP THE DATASET FOR ANALYSIS ---------------

#ANY BEHAVIORS DURING WALKS

LOS_dat$Any_Walk_Count<-
LOS_dat$Jumps_walk+LOS_dat$Grabs_walk+LOS_dat$Mouths_walk+LOS_dat$Vocalizes_
walk

LOS_dat$Any_Walk<-NA

LOS_dat$Any_Walk[LOS_dat$Any_Walk_Count>0]<-1

LOS_dat$Any_Walk[LOS_dat$Any_Walk_Count==0]<-0

table(LOS_dat$Any_Walk, LOS_dat$Any_Walk_Count)


#ANY BEHAVIORS DURING HARNESS

LOS_dat$Any_Harness_Count<-
LOS_dat$Jumps_harness+LOS_dat$Grabs_harness+LOS_dat$Mouths_harness+LOS_dat$
Vocalizes_harness

LOS_dat$Any_Harness<-NA

LOS_dat$Any_Harness[LOS_dat$Any_Harness_Count>0]<-1

LOS_dat$Any_Harness[LOS_dat$Any_Harness_Count==0]<-0

table(LOS_dat$Any_Harness, LOS_dat$Any_Harness_Count)


# FIXING QUEEN's ID

LOS_dat$Dog_ID[LOS_dat$Dog_ID=="32533"]<-"25532"

LOS_dat$Dog_ID<-droplevels(LOS_dat$Dog_ID)


LOS_dat %>% group_by(Dog_name,Dog_ID, Sex, Age_Class, Body_Size) %>%

 summarise(Jumps_harness_avg=mean(Jumps_harness),

      Jumps_walk_avg=mean(Jumps_walk),

      Grabs_harness_avg=mean(Grabs_harness),
```

```
        Grabs_walk_avg=mean(Grabs_walk),
        Mouths_harness_avg=mean(Mouths_harness),
        Mouths_walk_avg=mean(Mouths_walk),
        Vocalizes_harness_avg=mean(Vocalizes_harness),
        Vocalizes_walk_avg=mean(Vocalizes_walk),
        avg_pulls_walk=mean(total_pulls_walk),
        Any_Walk_Count_avg=mean(Any_Walk_Count),
        Any_Harness_Count_avg=mean(Any_Harness_Count),
        Length_of_stay=mean(Length_of_stay))->LOS_dat_agg

LOS_dat_agg$Jumps_harness <-NA
LOS_dat_agg$Jumps_harness[LOS_dat_agg$Jumps_harness_avg==0]<-0
LOS_dat_agg$Jumps_harness[LOS_dat_agg$Jumps_harness_avg>0]<-1
table(LOS_dat_agg$Jumps_harness)

LOS_dat_agg$Jumps_walk <-NA
LOS_dat_agg$Jumps_walk[LOS_dat_agg$Jumps_walk_avg==0]<-0
LOS_dat_agg$Jumps_walk[LOS_dat_agg$Jumps_walk_avg>0]<-1
table(LOS_dat_agg$Jumps_walk)

LOS_dat_agg$Grabs_harness <-NA
LOS_dat_agg$Grabs_harness[LOS_dat_agg$Grabs_harness_avg==0]<-0
LOS_dat_agg$Grabs_harness[LOS_dat_agg$Grabs_harness_avg>0]<-1
table(LOS_dat_agg$Grabs_harness)
```

```
LOS_dat_agg$Grabs_walk <-NA

LOS_dat_agg$Grabs_walk[LOS_dat_agg$Grabs_walk_avg==0]<-0

LOS_dat_agg$Grabs_walk[LOS_dat_agg$Grabs_walk_avg>0]<-1

table(LOS_dat_agg$Grabs_walk)


LOS_dat_agg$Mouths_harness <-NA

LOS_dat_agg$Mouths_harness[LOS_dat_agg$Mouths_harness_avg==0]<-0

LOS_dat_agg$Mouths_harness[LOS_dat_agg$Mouths_harness_avg>0]<-1

table(LOS_dat_agg$Mouths_harness)



LOS_dat_agg$Mouths_walk <-NA

LOS_dat_agg$Mouths_walk[LOS_dat_agg$Mouths_walk_avg==0]<-0

LOS_dat_agg$Mouths_walk[LOS_dat_agg$Mouths_walk_avg>0]<-1

table(LOS_dat_agg$Mouths_walk)


LOS_dat_agg$Vocalizes_harness <-NA

LOS_dat_agg$Vocalizes_harness[LOS_dat_agg$Vocalizes_harness_avg==0]<-0

LOS_dat_agg$Vocalizes_harness[LOS_dat_agg$Vocalizes_harness_avg>0]<-1

table(LOS_dat_agg$Vocalizes_harness)


LOS_dat_agg$Vocalizes_walk <-NA

LOS_dat_agg$Vocalizes_walk[LOS_dat_agg$Vocalizes_walk_avg==0]<-0

LOS_dat_agg$Vocalizes_walk[LOS_dat_agg$Vocalizes_walk_avg>0]<-1

table(LOS_dat_agg$Vocalizes_walk)
```

```
LOS_dat_agg$Any_Walk <-NA

LOS_dat_agg$Any_Walk[LOS_dat_agg$Any_Walk_Count_avg==0]<-0

LOS_dat_agg$Any_Walk[LOS_dat_agg$Any_Walk_Count_avg>0]<-1

table(LOS_dat_agg$Any_Walk)


LOS_dat_agg$Any_Harness <-NA

LOS_dat_agg$Any_Harness[LOS_dat_agg$Any_Harness_Count_avg==0]<-0

LOS_dat_agg$Any_Harness[LOS_dat_agg$Any_Harness_Count_avg>0]<-1

table(LOS_dat_agg$Any_Harness)


LOS_dat$Jumps_walk <-as.factor(LOS_dat$Jumps_walk)

LOS_dat$Jumps_harness <-as.factor(LOS_dat$Jumps_harness)

LOS_dat$Grabs_harness <-as.factor(LOS_dat$Grabs_harness)

LOS_dat$Grabs_walk <-as.factor(LOS_dat$Grabs_walk)

LOS_dat$Mouths_harness <-as.factor(LOS_dat$Mouths_harness)

LOS_dat$Mouths_walk  <-as.factor(LOS_dat$Mouths_walk)

LOS_dat$Vocalizes_harness <-as.factor(LOS_dat$Vocalizes_harness)

LOS_dat$Vocalizes_walk <-as.factor(LOS_dat$Vocalizes_walk)


LOS_dat_agg$Length_of_stay2<-LOS_dat_agg$Length_of_stay

LOS_dat_agg$Length_of_stay2[is.na(LOS_dat_agg$Length_of_stay)==T]<-112 #at the time of analysis, Remus was not adopted but had been at the shelter for 112 days



# Bivariate Analysis on Length of Stay

#Sex
```

```
LOS_dat_agg$Length_of_stay

LOS_dat_agg %>%
  group_by(Sex) %>%
  dplyr::summarise(mean_LOS=mean(Length_of_stay2, na.rm=T),
sd_LOS=sd(Length_of_stay2, na.rm=T), n=n())


t.test(log(Length_of_stay2)~ Sex, data=LOS_dat_agg)

cohens_d(log(Length_of_stay2)~ Sex, data=LOS_dat_agg)



#Age class

LOS_dat_agg %>%
  group_by(Age_Class) %>%
  summarise(mean_LOS=mean(Length_of_stay2, na.rm=T), sd_LOS=sd(Length_of_stay2,
na.rm=T), n=n())


ma1<-lm(log(Length_of_stay2)~ Age_Class, data=LOS_dat_agg)

anova(ma1)

eta_squared(ma1)

emmeans(ma1, revpairwise ~ Age_Class, type="response")



#Body Size

LOS_dat_agg %>%
  group_by(Body_Size) %>%
  summarise(mean_LOS=mean(Length_of_stay2, na.rm=T), sd_LOS=sd(Length_of_stay2,
na.rm=T), , n=n())
```

```
ma2<-lm(log(Length_of_stay2)~ Body_Size, data=LOS_dat_agg)

anova(ma2)

eta_squared(ma2)

emmeans(ma2, revpairwise ~ Body_Size, type="response")


#Jumps while walking

LOS_dat_agg %>%

  group_by(Jumps_walk) %>%

  summarise(mean_LOS=mean(Length_of_stay2, na.rm=T), sd_LOS=sd(Length_of_stay2, na.rm=T), , n=n())


t.test(log(Length_of_stay2)~ Jumps_walk, data=LOS_dat_agg)

cohens_d(log(Length_of_stay2)~ Jumps_walk, data=LOS_dat_agg)


#Grabs while walking

LOS_dat_agg %>%

  group_by(Grabs_walk) %>%

  summarise(mean_LOS=mean(Length_of_stay2, na.rm=T), sd_LOS=sd(Length_of_stay2, na.rm=T),, n=n())


t.test(log(Length_of_stay2)~ Grabs_walk, data=LOS_dat_agg)

cohens_d(log(Length_of_stay2)~ Grabs_walk, data=LOS_dat_agg)


#Mouths while walking

LOS_dat_agg %>%

  group_by(Mouths_walk) %>%
```

```
  summarise(mean_LOS=mean(Length_of_stay2, na.rm=T), sd_LOS=sd(Length_of_stay2,
na.rm=T),, n=n())

t.test(log(Length_of_stay2)~ Mouths_walk, data=LOS_dat_agg)

cohens_d(log(Length_of_stay2)~ Mouths_walk, data=LOS_dat_agg)

#Vocalizes while walking

LOS_dat_agg %>%

  group_by(Vocalizes_walk) %>%

  summarise(mean_LOS=mean(Length_of_stay2, na.rm=T), sd_LOS=sd(Length_of_stay2,
na.rm=T),, n=n())

t.test(log(Length_of_stay2)~ Vocalizes_walk, data=LOS_dat_agg)

cohens_d(log(Length_of_stay2)~ Vocalizes_walk, data=LOS_dat_agg)

#Jumps while harnessing

LOS_dat_agg %>%

  group_by(Jumps_harness) %>%

  summarise(mean_LOS=mean(Length_of_stay2, na.rm=T), sd_LOS=sd(Length_of_stay2,
na.rm=T),, n=n())

t.test(log(Length_of_stay2)~ Jumps_harness, data=LOS_dat_agg)

cohens_d(log(Length_of_stay2)~ Jumps_harness, data=LOS_dat_agg)

#Grabs while harnessing (rare event!)

LOS_dat_agg %>%

  group_by(Grabs_harness) %>%
```

```
  summarise(mean_LOS=mean(Length_of_stay2, na.rm=T), sd_LOS=sd(Length_of_stay2,
na.rm=T), n=n())

t.test(log(Length_of_stay2)~ Grabs_harness, data=LOS_dat_agg)

cohens_d(log(Length_of_stay2)~ Grabs_harness, data=LOS_dat_agg)

# Mouths while harnessing

LOS_dat_agg %>%

  group_by(Mouths_harness) %>%

  summarise(mean_LOS=mean(Length_of_stay2, na.rm=T), sd_LOS=sd(Length_of_stay2,
na.rm=T), n=n())

t.test(log(Length_of_stay2)~ Mouths_harness, data=LOS_dat_agg)

cohens_d(log(Length_of_stay2)~ Mouths_harness, data=LOS_dat_agg)

#Vocalizes while harnessing (rare event)

LOS_dat_agg %>%

  group_by(Vocalizes_harness) %>%

  summarise(mean_LOS=mean(Length_of_stay2, na.rm=T), sd_LOS=sd(Length_of_stay2,
na.rm=T), n=n())

t.test(log(Length_of_stay2)~ Vocalizes_harness, data=LOS_dat_agg)

cohens_d(log(Length_of_stay2)~ Vocalizes_harness, data=LOS_dat_agg)

# Average pulls per walk

summary(LOS_dat_agg$avg_pulls_walk)
```

```
hist(LOS_dat_agg$avg_pulls_walk)

cor.test(LOS_dat_agg$avg_pulls_walk, LOS_dat_agg$Length_of_stay2)

plot(Length_of_stay2~ avg_pulls_walk, data=LOS_dat_agg)



# Large Model with Behaviors and demographics


m1b <- lm(log(Length_of_stay2)~Sex + Age_Class+Body_Size+Jumps_walk +Grabs_walk+
Mouths_walk+Vocalizes_walk+ Jumps_harness +Mouths_harness+avg_pulls_walk,
data=LOS_dat_agg )

summary(m1b)

joint_tests(m1b)

eta_squared(m1b)

emmeans(m1b, revpairwise ~ Age_Class, type="response")

emmeans(m1b, revpairwise ~ Grabs_walk, type="response")

emmeans(m1b, revpairwise ~ Mouths_walk, type="response")

emmeans(m1b, revpairwise ~ Vocalizes_walk, type="response")
```
