# Supplementary material for: Behaviors of Shelter Dogs During Harnessing and Leash Walks: Prevalence, Demographics, and Length of Stay
Source: Animals (Basel). 2025 Mar 17;15(6):856. doi: 10.3390/ani15060856 (PMC11939286; doi:10.3390/ani15060856)
Supplement: Supplementary file 1 [file animals-15-00856-s001.zip › R scripts for Dog Demographics and Behavior.pdf]

```r
#This R file looks at the relationship between various dog behaviors and dog demographics


#Libraries

library(lme4)

library(lmerTest)

library(emmeans)

library(ggplot2)

library(car)

library(dplyr)

#Loading the data

behaviors_dat <- read.csv("dog characteristics and behaviors 11-14-2024.csv")


#Predictor variables \ Characteristics

behaviors_dat$Dog_ID<-as.factor(behaviors_dat$Dog_ID)

behaviors_dat$Sex<-as.factor(behaviors_dat$Sex)

behaviors_dat$Age_Class<-as.factor(behaviors_dat$Age_Class)

behaviors_dat$Body_Size<-as.factor(behaviors_dat$Body_Size)


behaviors_dat %>% group_by(Dog_ID) %>% filter(row_number()==1) ->First_walks


#ANY BEHAVIORS DURING WALKS

behaviors_dat$Any_Walk_Count<-behaviors_dat$Jumps_walk+behaviors_dat$Grabs_walk+behaviors_dat$Mouths_walk+behaviors_dat$Vocalizes_walk

behaviors_dat$Any_Walk<-NA

behaviors_dat$Any_Walk[behaviors_dat$Any_Walk_Count>0]<-1

behaviors_dat$Any_Walk[behaviors_dat$Any_Walk_Count==0]<-0
```

```
table(behaviors_dat$Any_Walk, behaviors_dat$Any_Walk_Count)


#ANY BEHAVIORS DURING HARNESS

behaviors_dat$Any_Harness_Count<-
behaviors_dat$Jumps_harness+behaviors_dat$Grabs_harness+behaviors_dat$Mouths_ha
rness+behaviors_dat$Vocalizes_harness

behaviors_dat$Any_Harness<-NA

behaviors_dat$Any_Harness[behaviors_dat$Any_Harness_Count>0]<-1

behaviors_dat$Any_Harness[behaviors_dat$Any_Harness_Count==0]<-0

table(behaviors_dat$Any_Harness, behaviors_dat$Any_Harness_Count)


behaviors_dat %>%filter(Age_Class %in% c(1,2))->behaviors_dat_young



# Jump While Harnessing

table(behaviors_dat$Jumps_harness)

prop.table(table(behaviors_dat$Jumps_harness))


behaviors_dat %>% group_by(Sex) %>% summarise(mean(Jumps_harness),
sum(Jumps_harness))

behaviors_dat %>% group_by(Age_Class) %>% summarise(mean(Jumps_harness),
sum(Jumps_harness))

behaviors_dat %>% group_by(Body_Size) %>% summarise(mean(Jumps_harness),
sum(Jumps_harness))


m1_jump<- glmer(Jumps_harness ~ Sex + Age_Class + Body_Size
+Walk_number+(1|Dog_ID)+(1|Walker_name), data=behaviors_dat, family="binomial",
control=glmerControl(optimizer="bobyqa"))
```

```
summary(m1_jump)

Anova(m1_jump)

emmeans(m1_jump, pairwise ~Age_Class, type="response" )

emmeans(m1_jump, pairwise ~Body_Size, type="response" )


# Jump While Walking

table(behaviors_dat$Jumps_walk)

prop.table(table(behaviors_dat$Jumps_walk))


behaviors_dat %>% group_by(Sex) %>% summarise(mean(Jumps_walk),
sum(Jumps_walk))

behaviors_dat %>% group_by(Age_Class) %>% summarise(mean(Jumps_walk),
sum(Jumps_walk))

behaviors_dat %>% group_by(Body_Size) %>% summarise(mean(Jumps_walk),
sum(Jumps_walk))


m2_jump<- glmer(Jumps_walk ~ Sex + Age_Class + Body_Size
+Walk_number+(1|Dog_ID)+(1|Walker_name),
data=subset(behaviors_dat,Age_Class!="4"), family="binomial",
control=glmerControl(optimizer="bobyqa"))

summary(m2_jump)

Anova(m2_jump)


emmeans(m2_jump, pairwise ~Age_Class, type="response" )

test(emmeans(m2_jump,  ~Age_Class), null=-7) #used to test test if significantly different
from 0, since age class 4 is not in the analysis

exp(-7)/(1-exp(-7))
```

```
# Grabs While Harnessing  (rare event, no model)

table(behaviors_dat$Grabs_harness)

prop.table(table(behaviors_dat$Grabs_harness))


behaviors_dat %>% group_by(Sex) %>% summarise(mean(Grabs_harness),
sum(Grabs_harness))

behaviors_dat %>% group_by(Age_Class) %>% summarise(mean(Grabs_harness),
sum(Grabs_harness))

behaviors_dat %>% group_by(Body_Size) %>% summarise(mean(Grabs_harness),
sum(Grabs_harness))


#Grabs While Walking (rare event, no model)

table(behaviors_dat$Grabs_walk)

prop.table(table(behaviors_dat$Grabs_walk))


behaviors_dat %>% group_by(Sex) %>% summarise(mean(Grabs_walk), sum(Grabs_walk))

behaviors_dat %>% group_by(Age_Class) %>% summarise(mean(Grabs_walk),
sum(Grabs_walk))

behaviors_dat %>% group_by(Body_Size) %>% summarise(mean(Grabs_walk),
sum(Grabs_walk))


# Mouths while Harnessing (rare event, no model)


table(behaviors_dat$Mouths_harness)

prop.table(table(behaviors_dat$Mouths_harness))
```

```
behaviors_dat %>% group_by(Sex) %>% summarise(mean(Mouths_harness), sum(Mouths_harness))

behaviors_dat %>% group_by(Age_Class) %>% summarise(mean(Mouths_harness), sum(Mouths_harness))

behaviors_dat %>% group_by(Body_Size) %>% summarise(mean(Mouths_harness), sum(Mouths_harness))


# Mouths While Walking (rare event, no model)

table(behaviors_dat$Mouths_walk)

prop.table(table(behaviors_dat$Mouths_walk))


behaviors_dat %>% group_by(Sex) %>% summarise(mean(Mouths_walk), sum(Mouths_walk))

behaviors_dat %>% group_by(Age_Class) %>% summarise(mean(Mouths_walk), sum(Mouths_walk))

behaviors_dat %>% group_by(Body_Size) %>% summarise(mean(Mouths_walk), sum(Mouths_walk))


# Vocalizes During Harnessing (rare event, no model)


table(behaviors_dat$Vocalizes_harness)

prop.table(table(behaviors_dat$Vocalizes_harness))


behaviors_dat %>% group_by(Sex) %>% summarise(mean(Vocalizes_harness), sum(Vocalizes_harness))

behaviors_dat %>% group_by(Age_Class) %>% summarise(mean(Vocalizes_harness), sum(Vocalizes_harness))

behaviors_dat %>% group_by(Body_Size) %>% summarise(mean(Vocalizes_harness), sum(Vocalizes_harness))
```

```
# Vocalizes during walk

table(behaviors_dat$Vocalizes_walk)

prop.table(table(behaviors_dat$Vocalizes_walk))


behaviors_dat %>% group_by(Sex) %>% summarise(mean(Vocalizes_walk), sum(Vocalizes_walk))

behaviors_dat %>% group_by(Age_Class) %>% summarise(mean(Vocalizes_walk), sum(Vocalizes_walk))

behaviors_dat %>% group_by(Body_Size) %>% summarise(mean(Vocalizes_walk), sum(Vocalizes_walk))


vocal_walk<- glmer(Vocalizes_walk ~ Sex + Age_Class + Body_Size+Walk_number +(1|Dog_ID)+(1|Walker_name), data=behaviors_dat, family="binomial", control=glmerControl(optimizer="bobyqa"))

summary(vocal_walk)

Anova(vocal_walk, type=3)

emmeans(vocal_walk, consec ~Sex, type="response" )



# Total Pulls during Walk

behaviors_dat %>% summarise(mean(total_pulls_walk), sd(total_pulls_walk))

hist(behaviors_dat$total_pulls_walk)


behaviors_dat %>% group_by(Sex) %>% summarise(mean(total_pulls_walk), sd(total_pulls_walk))
```

```
behaviors_dat %>% group_by(Age_Class) %>% summarise(mean(total_pulls_walk),
sd(total_pulls_walk))

behaviors_dat %>% group_by(Body_Size) %>% summarise(mean(total_pulls_walk),
sd(total_pulls_walk))


total_walks<- glmer.nb(total_pulls_walk ~ Sex + Age_Class + Body_Size
+Walk_number+(1|Dog_ID)+(1|Walker_name), data=behaviors_dat)

summary(total_walks)

Anova(total_walks, type=3)

emmeans(total_walks, revpairwise ~Body_Size, type="response" )

emmeans(total_walks, revpairwise ~Age_Class, type="response" )


# Any Behaviors during Harnessing

table(behaviors_dat$Any_Harness)

prop.table(table(behaviors_dat$Any_Harness))


behaviors_dat %>% group_by(Sex) %>% summarise(mean(Any_Harness),
sum(Any_Harness))

behaviors_dat %>% group_by(Age_Class) %>% summarise(mean(Any_Harness),
sum(Any_Harness))

behaviors_dat %>% group_by(Body_Size) %>% summarise(mean(Any_Harness),
sum(Any_Harness))


table(behaviors_dat$Any_Harness, behaviors_dat$Jumps_harness)

table(behaviors_dat$Any_Harness, behaviors_dat$Grabs_harness)

table(behaviors_dat$Any_Harness, behaviors_dat$Mouths_harness)

table(behaviors_dat$Any_Harness, behaviors_dat$Vocalizes_harness)
```

```
any_model1<- glmer(Any_Harness ~ Sex + Age_Class + Body_Size+Walk_number
+(1|Dog_ID)+(1|Walker_name), data=behaviors_dat, family="binomial",
control=glmerControl(optimizer="bobyqa"))

summary(any_model1)

Anova(any_model1, type=3)

emmeans(any_model1, consec ~Sex, type="response" )

emmeans(any_model1, pairwise ~Age_Class, type="response" )



#Any Behaviors during walking

table(behaviors_dat$Any_Walk)

prop.table(table(behaviors_dat$Any_Walk))


behaviors_dat %>% group_by(Sex) %>% summarise(mean(Any_Walk), sum(Any_Walk))

behaviors_dat %>% group_by(Age_Class) %>% summarise(mean(Any_Walk),
sum(Any_Walk))

behaviors_dat %>% group_by(Body_Size) %>% summarise(mean(Any_Walk),
sum(Any_Walk))


table(behaviors_dat$Any_Walk, behaviors_dat$Jumps_walk)

table(behaviors_dat$Any_Walk, behaviors_dat$Grabs_walk)

table(behaviors_dat$Any_Walk, behaviors_dat$Mouths_walk)

table(behaviors_dat$Any_Walk, behaviors_dat$Vocalizes_walk)


any_model1<- glmer(Any_Walk ~ Sex + Age_Class + Body_Size+Walk_number
+(1|Dog_ID)+(1|Walker_name), data=behaviors_dat, family="binomial",
control=glmerControl(optimizer="bobyqa"))

summary(any_model1)
```

```
Anova(any_model1, type=3)

emmeans(any_model1, pairwise ~Age_Class, type="response" )
```
